# Yields, chemical composition, and antimicrobial activity of two Algerian essential oils against 40 avian multidrug-resistant *Escherichia coli* strains

**DOI:** 10.14202/vetworld.2018.1539-1550

**Published:** 2018-11-06

**Authors:** Narimene Mansouri, Leila Aoun, Nabila Dalichaouche, Douniazed Hadri

**Affiliations:** 1 Laboratory Research of Epidemiologic Monitoring, Health, Production, Reproduction, Experimentation and Cellular Therapy of Domestic and Wild Animals, Department of Veterinary Medicine, University of Chadli Bendjedid, El-Tarf, Algeria; 2 Regional Veterinary Laboratory of El-Tarf, National Institute of Veterinary Medicine, Minister of Agriculture, Algeria; 3 Department of Veterinary Medicine, University of Chadli Bendjedid, El-Tarf, Algeria

**Keywords:** Avian *Escherichia coli*, antimicrobial resistance, essential oils, antibacterial activity, *Thymus vulgaris* L, *Coriandrum sativum* L

## Abstract

**Aim:**

The aim of this study is to investigate, *in vitro*, a possible antibacterial activity of Algerian essential oils (EOs) of Thyme (*Thymus vulgaris* L.) and that of Coriander (*Coriandrum sativum* L.) against multidrug-resistant avian *Escherichia coli* strains and this in a perspective of their future use as a substitute for antibiotics (ATBs).

**Materials and Methods:**

In addition to the reference strain of *E. coli* ATCC 25922, 40 strains of avian *E. coli* have been isolated (24 strains of broilers and 16 of turkeys), their antimicrobial resistance profile was determined by antibiogram tests against 21 ATBs whereupon they were subjected to the action of two Algerian EOs; the EO of Thyme (*T. vulgaris* L.) and that of Coriander (*C. sativum* L.), which oils were extracted by hydrodistillation and analyzed by Gas Chromatography coupled to Mass Spectrometry (GC-MS) and this for the determination of their chemical composition. The antibacterial activity, resulting in zones of inhibition, was evaluated by carrying out, in triplicate, aromatograms for both pure EO and that which has been diluted to 15% in Dimethyl Sulfoxide (DMSO), while the minimum inhibitory concentrations (MIC) of the two EOs were highlighted by the method of liquid macrodilution.

**Results:**

Antibiogram performance demonstrated an alarming state of antimicrobial resistance, the multidrug resistance rate was estimated at 100% for the broilers chicken strains and at 81.25% for strains isolated from turkeys, hydrodistillation allowed to obtained EOs with yields estimated at 1.22±0.26% for Thyme EO and 0.23±0.15% for the essence of Coriander, the GC-MS analysis identified 19 main compounds and showed that the majority chemical components were Carvacrol (73.03%) for Thyme volatile oil and Linalool (60.91%) for Coriander EO, aromatograms and the determination of MIC concluded that the EO of Thyme showed a greater antibacterial activity with an average of the zones of inhibition estimated at 26.75±0.426 mm and MIC ranging from 0.07 to 0.93 mg/ml against an average of the inhibition zones evaluated at 17.05±0.383 mm and MICs evaluated between 0.6 and 10 mg/ml for the EO of Coriander.

**Conclusion:**

In aviculture, these results seem to be very promising in the case where we think about the replacement of ATBs by EOs, *in vivo* studies would be very interesting to confirm or invalidate this hypothesis.

## Introduction

In veterinary medicine and particularly in poultry farming, antimicrobial resistance is a real public health problem; the anarchic use of antibiotics (ATBs) has led to the development of bacteria with an alarming profile of resistance [1]. In Algeria, as in the majority of developing countries, the poultry sector is one of the most prosperous sectors, and white meat is the most consumed meat, so any selection of ATB resistance will inevitably affect the health of Algerian consumers [2].

In recent years, the number of scientific articles relating to aromatherapy and demonstrating its benefits has increased [3], that is why we naturally have opted, in our study, for the use of two essential oils (EOs) that of Thyme (*Thymus vulgaris* L.) and Coriander (*Coriandrum sativum* L.). These aromatic and medicinal plants are widely used, and their benefits have been recognized since Antiquity [4]. In fact, Thyme, widespread in Algeria, constitute an important remedy used since centuries and identified as a good source of bioactive compounds possessing significant antioxidant and anti-inflammatory properties, potentially effective in prevention and treatment of pathological conditions [5], but what prompted us the most to choose it is its reputation of antibacterial molecule, especially against Enterobacteria, one of the most important bacteria in avian pathology [6]. Coriander, another aromatic plant common in the Mediterranean basin, also has EOs known for their antifungal, insecticides, and antibacterial effects [7,8].

Following this observation, our approach was to think about a new substances that could have, *in vitro*, the same antibacterial effect as that of ATBs, while having the advantage of avoiding the selection of new resistant bacteria, which is why, we thought to an ancient cure, namely aromatic plants and especially their EOs [9]. Our approach was, therefore, to look for the antibacterial action of these EO to remedy the evolution of antimicrobial resistance, thus providing consumers with healthy foodstuffs.

## Materials and Methods

### Ethical approval

Ethical approval was not required in this study since no live animals were used in the experiment. Samples were collected from dead animals.

### Plant material

Dried aerial parts (stems, leaves, and flowers) for Thyme EO and ground seeds for Coriander EO were purchased from a herbalist in the city of El-Tarf (Northeastern Algeria), these plants come from the summer harvest (Flowering season) of the year 2015 in the region of Djelfa located in the central part of Northern Algeria which is a region characterized by a semi-arid climate.

### Extraction and isolation of EOs

Extraction of the EOs was carried out by hydrodistillation using the Deryng apparatus which is the Polish version of the Clevenger apparatus [10], several distillations were carried out by boiling 100 g of dried aerial parts of Thyme in 1 L of distilled water [11] and 30 g of ground Coriander in 500 ml of distilled water during 3 h, the yield of EO was determined in relation to the dry matter [10]. After decanting and drying of the oils over anhydrous sodium sulfate, they were stored at 4°C in amber glass tubes and the dark until analysis [12].

### Gas Chromatography-Mass Spectrometry (GC-MS) analysis

The chemical composition analysis of EOs of Thyme (*T. vulgaris*) and that of Coriander (*C. sativum* L.) was performed in Algiers in the Center for Scientific and Technical Research in Physical and Chemical Analysis (CRAPC EXPERTISE SPA) using g a Hewlett Packard Agilent 6890 GC equipped with an HP-5MS capillary column (30 m * 0.25 mm, film thickness 0.25 μm). The steady state temperature was started at 60 °C for 8 min and then gradually increased (2 °C/min) to 250 ° C for 10 min. This device was coupled to a Hewlett Packard Agilent 5973 MS detector recorded in 70 eV electron ionization with 30 to 550 sweep with a solvent delay of 3.5 min, ion source at 230 °C and interface temperature at 280 °C. The temperature of the injector was fixed to 250°C with a split ratio of 50:1 and 0.2 µl of injected volume. Helium was used as the carrier gas at a flow rate of 0.5 ml/. The percentage of each constituent of the oil was determined by area peaks, the EOs components were identified by comparison with literature data and the profiles from the Wiley 7.

### Bacterial strains

In our study, the bacteria we worked on were represented by 40 multidrug-resistant strains of avian *Escherichia coli* (24 strains from broiler chicken and 16 from turkey) isolated from animals reared in the Eastern region of Algeria and this from feces, livers, and intestines of birds with diarrhea. These samples were restored in buffered peptone water for 18-24 h at 37°C, then, using a sterile platinum loop, a drop from the previously inoculated broth was seeded onto Hektoen agar plates for 24 h at 37°C. Identification of *E. coli* isolates was performed according to morphological characters of colonies and results of their biochemical tests obtained using commercial biochemical test kits (bioMérieux API, France). We also worked on a reference strain of *E. coli* (ATCC 25922) provided by the Regional Veterinary Laboratory of El-Tarf.

### Evaluation of resistance phenotypes of E. coli avian strains

The resistance profile of isolated strains was studied by performing antibiograms (antimicrobial susceptibility testing) according to the recommendations of the Antibiogram Committee of the French Society of Microbiology (CA-SFM - Veterinary Antibiograms - version 2017). These antibiograms were carried out against 21 ATBs belonging to 6 ATB families (Table-1) and for each group of antibiograms performed, internal quality control was done with the *E. coli* reference strain (ATCC 25922), and this for ensuring the validity of the results of the antibiograms obtained [13].

**Table-1:** Families of antibiotics used.

Antibiotic family	Subfamily	Molecule	Concentration	Disc initials
Aminoglycosides	-	Gentamicin	15 µg	GM
		Neomycin	30 UI	NEO
Beta-lactams	Aminopenicillins	Amoxicillin/clavulanic acid	(20/10 µg)	AMC
		Ampicillin	(10 µg)	AMP
	Carbapenems	Ertapenem	(10 µg)	ETP
		Imipenem	(10 µg)	IMI
	Cephalosporins first generation	Cephalothin	(30 µg)	CF
	Cephalosporins second generation	Cefoxitin	(30 µg)	FOX
	Cephalosporins third generation	Cefotaxime	(30 µg)	CTX
		Ceftazidime	(30 µg)	CAZ
		Ceftiofur	(30 µg)	XNL
		Ceftriaxone	(30 µg)	CRO
Quinolones	Cephalosporins fourth generation	Cefepime	(30 µg)	FEP
	Monobactams	Aztreonam	(30 µg)	AT
	first generation	Nalidixic acid	(30 µg)	NA
	Fluoroquinolones second generation	Ciprofloxacin	(5 µg)	CIP
	Fluoroquinolones third generation	Danofloxacin	(5 µg)	DAN
		Enrofloxacin	(5 µg)	ENR
Polymyxins	Polypeptide	Colistin	(50 µg)	CS
Tetracyclines	-	Tetracycline	(30 UI)	TE
Trimethoprim/sulfonamides	-	Trimethoprim/Sulfamethoxazole	(1.25/23.75 µg)	SXT

### Disc diffusion assay

This method, also called aromatogram, is based on a technique used in medical bacteriology which is the antibiogram, the only difference is the replacement of ATBs with aromatic extracts, the EO will diffuse from the soaked disc within the agar and determine a concentration gradient [14]. For this, a bacterial suspension of density equivalent to 0.5 MacFarland is prepared and diluted 1/100, in the meantime, 20 ml of Mueller-Hinton agar medium are prepared and poured into a Petri dish where 2 ml of inoculum are poured, after impregnation of 5 min, the excess inoculum is removed by suction. On the surface of each box, three sterile 6 mm diameter filter paper disks (bioMérieux) are deposited. Two tests are carried out: A disc soaked with 15 μl of EO and the second one with 15 μl of EO supplemented with 15% of Dimethyl Sulfoxide (DMSO). A negative control is carried out with 15 μl of sterile distilled water in the presence of 15% of DMSO. The dishes are left for 1 h at room temperature, then inverted and incubated at 37°C for 18-24 h [15]. The bacteria will grow on the entire surface of the agar except where they meet a concentration of EO sufficient to inhibit their growth. At the outlet of the stove, the absence of bacterial growth results in a translucent halo around the disc and the diameter of the inhibition zone (IZ) is measured and expressed in millimeters (mm). The larger the diameter, the more the strain is sensitive to EO [14]. Each test was performed in triplicate.

### Determination of Minimum Inhibitory Concentration (MIC)

In this step, we drew on the work of Dr. Guinoiseau using the method of liquid macrodilution for the determination of MICs, and this method consists of inoculating, by a standardized inoculum and a decreasing concentration range in EO. After incubation, observation of the range gives access to the MIC [15], which corresponds to the lowest concentration of active ingredient capable of inhibiting any bacterial growth at 24 h [3].

## Results and Discussion

### Yields and organoleptic properties

Extraction by several hydrodistillations of the two plants allowed to obtain different yields. Indeed, the EO of *T. vulgaris* L., characterized by a liquid appearance, a light brown color, a strong and a spicy aromatic odor, expressed a yield estimated at 1.22±0.26%, while the EO of *C. sativum* L., which was characterized by its mobile liquid appearance, pale yellow, and its camphorous odor, displayed, as for her, a much lower yield evaluated at 0.23±0.15%. Table-2 compares the yields we obtained with several other studies [16-33], carried out on the same plants, in Algeria, Maghreb, Africa, and other regions of the world.

**Table-2:** Comparison of the yields obtained with the data from the literature.

EO	Yields

	Present study (%)	Literature data (%)			

		Algerian studies	Maghrebian studies	African studies	Other studies
TνEO	1.22±0.26	0.45-2.7 [16,17]	1-3.6 [18-20]	0.55-1 [21,22]	0.83-4 [23-25]
C*s*EO	0.23±0.15	0.44-0.70 [26]	0.34 [27]	0.31-0.8 [28,29]	0.15-2.1 [30-33]

EO=Essential oil, TνEO=*Thymus vulgaris* L. Essential oil, *CsEO=Coriandrum sativum* L. Essential oil

The results illustrated in Table-2 demonstrate a certain variability in the yields obtained in the various studies conducted [16-33]; indeed, these differences could be explained by several factors such as environmental factors as demonstrated by Krol and Kieltyka-Dadasiewicz [34] who emphasized that the weather conditions and harvested time have a significant effect on the herb yield, in the same context, Jordan *et al*. [35] gave a relationship between the production of volatile oils and the climate by stating that EO production is favored in areas with a low thermicity index. Other studies examining the influence of plant maturity on their EO yield have shown that higher yields have been recorded on advanced mature plants [27]. Hazzoumi *et al*. [36], when to them, have highlighted a negative correlation between the water content in plant leaves and their EO yields, while other authors have attributed the difference of these yields to the plant drying time [37] and to the means of EO extraction, Indeed, it has been proven, especially by Akram *et al*. [38], where EO extraction was done by two different methods, a supercritical fluid extraction (SCFE) and hydrodistillation, that the yield of the EO differs according to the extraction technique. The comparison between these two methods revealed the superiority of the yields obtained by SCFE compared to the hydrodistillation process. While Zheljazkov *et al*. [39] emphasized the duration of hydrodistillation by demonstrating that the low EO yield obtained in the short distillation time (DT) has been increased with increasing of this DT, the extraction temperature and the flow rate can also be incriminate [40]. Other parameters such as growing region [35], geographical variations, and origin of cultivars also appear to be behind the variations in these yields [41].

### Chemical composition of the EOs

The GC-MS analysis of the essences studied gave the following results (Table-3), for Thyme EO (*T. vulgaris* L.), 14 compounds, representing 95.58% of all the constituents detected, were identified. This EO was characterized by its high content of phenols (74.40%). Indeed, among the 7 main constituents of this aroma, the main compound was Carvacrol (73.03%) which is a powerful phenol sought in EOs for its antibacterial action, which molecule was followed by p-cymen (9.99%), β-caryophyllene (3.63%) and γ-terpinene (3.02%), which together with p-cymen represent the two precursors of Carvacol [42,43], note that Thymol, another potent phenol is present but in small quantities (1.14%). For Coriander EO (*C. sativum*), GC-MS analysis revealed 19 molecules representing 93.51% of the total components; the main molecule was Linalool (60.91%), followed by Eugenol (8.95%) and Aceteugenol (6.70%). The chemical analysis of these essences allowed, therefore, highlighting 2 different chemotypes (CT), the CT Carvacrol for the EO of Thyme and Linalool CT for EO of Coriander.

**Table-3:** Chemical composition of the essential oils studied (*Thymus vulgaris* L. and *Coriandrum sativum* L.).

Compounds	RT (min)	% Content	

		TνEO	C*s*EO
α-Pinene	9.142	0.55	2.52
Camphene	9.879	0.14	0.23
β-Pinene	11.610	0.06	0.28
β-Myrcene	12.477	0.95	0.29
α-Terpinene	14.124	1.24	0.12
p-Cymene	14.570	9.99	1.05
γ-Terpinene	17.546	3.02	3.25
Linalool	20.313	1.17	60.91
Camphor	23.311	-	1.98
Borneol	24.906	0.41	0.07
α-Terpineol	26.743	-	0.32
Decanal	27.578	-	1.07
Geraniol	31.246	-	1.34
Thymol	35.055	1.14	0.36
Carvacrol	36.371	73.03	1.20
Eugenol	38.197	0.23	8.95
Geranyl acetate	39.593	-	2.19
Trans (β) caryophyllene	41.543	3.63	0.68
Aceteugenol	48.376	0.02	6.70
Chemical group		15.95	9.08
Monoterpene hydrocarbons (%)		74.40	10.51
Phenols (%)		01.58	63.28
Oxygenated monoterpenes (%)		03.63	0.68
Sesquiterpenes hydrocarbons (%)		0.02	09.96
Others (%)		98.58	93.51
Total (%)		

RT=Retention time, TνEO=*Thymus vulgaris* L. Essential oil, C*s*EO=*Coriandrum sativum* L. Essential oil

By analyzing our results and comparing them with other studies (Table-4) [16,17,19,26,28,31,32,44-46], we can make the following observations; concerning the EO of Thyme, the majority components are the same as we obtained but with different levels allowing to have other CT. Indeed, apart Sidali *et al*. [16], who worked on *T. vulgaris* L. from Northwest Algeria and whose results attributed, as for us, their EO to the Carvacrol CT, the other studies presented other CT. The study carried in Algeria (North Center) [17] and on the same plant, displayed an EO belonging to the Linalool CT in Morocco [19], in Italy [31] and in Iran [44], have highlighted an EO with Thymol CT. In Romania, another CT, the γ-terpinene one, has been reported [45]. Note that in the literature, for the EO of *T. vulgaris* L., there are up to 20 different CTs [47].

**Table-4:** Comparison of the chemical compositions obtained with the data from the literature.

*Thymus vulgaris* L. Essential Oil		

Origin	Major components	References
Our study	Carvacrol (73.03), *p*-Cymen (9.99), Caryophyllene (3.6), γ-Terpinene (3.02), Linalool (1.17), Thymol (1.14)	-
Algeria (Northwest)	Carvacrol (55.2), γ-Terpinene (12.6), *p*-Cymen (9.3), Linalool (3.9)	[16]
Algeria (North Center)	Linalool (82.88), Thymol (4.92), Linalyl acetate (2.43), Cymene (2.08)	[17]
Morocco	Thymol (35.8), Carvacrol (18.6), *p*-Cymen (14.1), γ-Terpinene (12.8)	[19]
Italy	Thymol (43.68), *p*-Cymen (18.5), Carvacrol (5.5), γ-Terpinene (4.9)	[31]
Iran	Thymol (40.02), Carvacrol (18.31), *p*-Cymen (16.78), Linalool (4.84)	[44]
Romania	γ-Terpinene (68.41), Thymol (24.72), Caryophyllene (5.50)	[45]

***Coriandrum sativum*** L. Essential Oil			

**Origin**	**Major components**	**References**	

Our study	Linalool (60.91), Eugenol (8.95), Aceteugenol (6.70),γ-Terpinene (3.25)	-
Algeria (Northwest)	Linalool (63.50), Camphor (2.69), Geraniol (1.79), Limonene (0.29)	[26]
Egypt (North)	Linalool (59.6), Ethyl hexanoic acid (4.9), Sabinene,(4.36) α-Thujene (3.32)	[28]
Italy	Linalool (77.07), Geraniol (5.24), Caryophyllene (3.16), Camphor (2.60)	[31]
Syria	Linalool (73.92), Geranyl acetate (4.43), o-Cymen (2.39)	[32]
Egypt (Center)	Linalool (70.93), Linalool acetate (4.78), -Pinene (4.17), *p*-Cymen (3.63)	[46]

As for the EO of *C. sativum* L., the result that we obtained is corroborated by several other studies carried out in Algeria (Northwest) [26], Egypt (North and Center) [28,46], Italy [31], and Syria [32], thus affirming the belonging to the Linalool CT. The variations reported in the compositions of the EO studied can be explained by intrinsic plant factors and extrinsic one [48]; these factors can be divided into three groups; genotype, ecological, and technological factors [49-51]. instead of [49-51], in fact, Pirbalouti [52], during his work, indicated that the components of EOs varied with the plant genotype, El-Zaeddi *et al*. [53], when to them, have rather questioned the stage of maturity of the plant, while Luís *et al*. [54] have instead focused on the geographical origin of the plant; however, the environmental factor is also important. Indeed, Atti-Santos *et al*. [55], working on the effect of the seasonal variation of the chemical composition of the EO of *T. vulgaris* from South Brazil, found that the main component, namely Thymol, over the 9 months of harvesting period, was found to vary from 35.5% to 52.4%, concluding that as regard to the harvest time, EO was richer in oxygenated compounds in spring season. Other authors have attributed the difference in the chemical composition of EO to the conditions in which the plants will grow, thus calling into question the temperature, photoperiod, rainfall, and hygrometry [56-58], abiotic stress such as salinity and water stress [32], agronomical practices [59], and even spatial distribution of the plants. Indeed, De Falco *et al*. [60], in addition to incriminating the state of the plant before extraction (fresh or dried), demonstrated an EO rich in Sabinene from plants grown in single rows, while plants grown in double rows were richer in Ocimene. Finally, other factors, influencing the composition of EO, can be mentioned such as the period and conditions of storage [61], the duration of drying of the plant, as well as the extraction duration [37].

### ATBs resistance of E. coli avian strains

The results of ATB resistance of *E. coli* strains are presented in Table-5; for the broiler chicken strains, antibiograms showed a very high and very disturbing antimicrobial resistance profile. Indeed, of the 21 ATBs tested, a total resistance (100%), combining the intermediate and resistant antibiogram results (I+R), has been reported to 8 molecules; amoxicillin/clavulanic acid (AMC), ampicillin (AMP), ceftazidime (CAZ), which is a cephalosporin third generation, nalidixic acid (NA), danofloxacin (DAN), enrofloxacin (ENR), tetracycline (TE), and trimethoprim/sulfamethoxazole (SXT), these strains showed also a significant resistance (I+R) to colistin (CS) (95.83%), ciprofloxacin (CIP) (87.5%) and neomycin (NEO) (75%); note that the cefepime (FEP), cephalosporin fourth generation, is the only ATB for which no antimicrobial resistance has been reported (0%), but what also attracted our attention is the presence of a resistance to two molecules belonging to the carbapenem family, namely ertapenem (ETP) and imipenem (IMI), evaluated respectively at 8.33 and 4.16%. although this resistance is minimal, it is very worrying as this class of ATB ranked as critically important by World Health, is used in hospitals as a treatment of last intention [62]. Unfortunately, the result we obtained is corroborated by several other studies conducted in Algeria; this is the case of Halfaoui *et al*. [63] who worked on different organs of broilers with colibacillosis lesions in central Algeria. This study isolated 156 strains of *E. coli* with a high level of resistance to TE (94.12%), flumequine (FLM): 91.5%, SXT (88.89%), ENR (86.27%), NA (85.62%), AMP (83.01%) and doxycycline (DO): 75.81%. Another study, conducted on ATB resistance of avian Enterobacteriaceae in Western Algeria by Ahmed *et al*. [64], found that *E. coli* strains presented a high levels of resistance to FLM (94%), TE, and amoxicillin with the same rate estimated at 92%, SXT (91%), ENR (86%), NA (84%), and cephalothin (CF) with 80% of resistance; a second study, conducted in another region of the West region of Algeria, also revealed high antimicrobial resistance levels for *E. coli* from broilers with up to 90.35%, 79.82%, 70.17%, 92.10%, and 62.28%, respectively, for TE, ENR, SXT, AMC, and ceftiofur (XNL) [65]. Other similar results were recorded on *E. coli* strains from broiler in other countries; Abd El Tawab *et al*. [66] showed *E. coli* strains with total resistance (100%) to AMC and significant resistance to 2 other ATB which are DO and erythromycin (ERY) with, respectively, rates estimated at 90% and 60% in Egypt; Manishimwe *et al*. [67] reported a prevalence of ATB resistance (I+R) from *E. coli* isolates to ERY, rifampicin, and DO estimated, respectively, at 100, 98.8, and 98.3% in Rwanda, while Wasyl *et al*. [68] estimated at 54.5% the resistance of *E. coli* to cephalosporin in Poland; these genes were also noted in strains from broilers originated from Belgium and Germany.

**Table-5:** Antibiograms results of avian *E. coli* strains.

Antibiotics	% Resistance BS (I+R)	% Resistance TS (I+R)
GM	8.33	6.25
NEO	75	81.25
AMC	100	87.5
AMP	100	81.25
ETP	8.33	6.25
IMI	4.16	00
CF	54.16	56.25
FOX	45.83	50
CTX	12.5	6.25
CAZ	100	81.25
XNL	29.16	12.5
CRO	4.16	12.5
FEP	00	6.25
AT	4.16	00
NA	100	87.5
CIP	87.5	75
DAN	100	87.5
ENR	100	87.5
CS	95.83	100
TE	100	81.25
SXT	100	68.75
% of multi-resistant isolates	100	81.25

ATB=Antibiotics, BS=Broiler strains, (I+R)=Intermediate+resistant, TS=Turkey strains, GM=Gentamicin, NEO=Neomycin, AMC=Amoxicillin/clavulanic acid, AMP=Ampicillin, ETP=Ertapenem, IMI=Imipenem, CF=Cephalothin, FOX=Cefoxitin, CTX=Cefotaxime, CAZ=Ceftazidime, XNL=Ceftiofur, CRO=Ceftriaxone, FEP=Cefepime, AT=Aztreonam, NA=Nalidixic acid, CIP=Ciprofloxacin, DAN=Danofloxacin, ENR=Enrofloxacin, CS=Colistin, TE=Tetracycline, SXT=Trimethoprim/sulfamethoxazole, *E. coli=Escherichia coli*

However, our results differ completely from those obtained in France and expressed in the annual report of the year 2016 of the network RESAPATH (French surveillance network for antimicrobial resistance in pathogenic bacteria of animal origin) in collaboration with ANSES (French agency for food, environmental, and occupational health safety), in fact, this network reported that the resistance to TE in clinical *E. coli* broiler strains has been continuously decreasing from 81% in 2010 to 44% in 2016. The same thing was found for XNL, where its resistance decreased from 22% in 2010 to 2.4% in 2016, and this network also reported a sensitivity of these *E. coli* strains with regard to ENR (92%), DAN (87%), AMC (85%), SXT (69%), and NA (53%) [69,70]. Regarding the CS, apart from the study carried out by Bodering *et al*. [71] where the resistance rate of *E. coli* isolated at CS was estimated at 100%, the majority of the other studies showed resistance rates at the CS that did not exceed 16% [72,73], or even a total sensitivity to this molecule for some studies [69].

In the case of strains isolated from turkeys, the finding is a significant antimicrobial resistance but less than that of broilers; in fact, a state of resistance (I + R) evaluated at 100%, has been demonstrated for a single molecule (CS) which, according to a study by Messai [74], is the first molecule to be used in Algeria in the Poultry farming during digestive infections. These bacteria also showed an estimated resistance of 87.5% against 4 molecules (AMC, NA, DAN and ENR) followed closely by a resistance rate evaluated at 81.25% recorded against four other molecules (NEO, AMP, CAZ and TE). The CIP, SXT, CF, and the cefoxitin came after with, respectively, posted rates at 75, 68.75, 56.25, and 50%. For the three ATB (IMI, ceftriaxone, and aztreonam), no antimicrobial resistance was reported (0%). We would like to point out that for these strains of turkey, the state of non-sensitivity to an ATB of the Carbapenem family, namely, ETP (6.25%) as well as the presence of resistance against a fourth-generation Cephalosporin, the FEP (6.25%), the only ATB, as previously mentioned, for which no resistance has been recorded in *E. coli* from broiler chicken, is a real public health problem, especially considering that turkey farming in Algeria is a very recent sector compared to broiler farming.

Gosling *et a*l. [75], working on CIP resistance in *E. col*i isolated from turkeys, recorded a rate of multi-resistant isolates estimated at 88.1% with resistances, 100% for both CIP and NA, 94.4% for TE, and 84.9% for AMP in Great Britain; the only result that does not agree with ours is the sensitivity of these strains to AMC (92.8%).

Our results are completely contradictory with those highlighted in France by the RESAPATH network which is a report of a decline in antimicrobial resistance and an increase in the sensitivity of *E. coli* strains from turkey meat to ATB; indeed, a significant increase in the proportion of susceptible isolates to CS was observed in all animal species what suggest that the spread of CS-resistant *E. coli* that is pathogenic for animals is under control; for TE, the sensitivity increased from 17% in 2010 to 58% in 2016, and the same thing for FLM which went from 66% in 2010 to 78% in 2016; this network also has a sensitivity state estimated at 99% for the NEO, 97% for the CF, 95% for the ENR, and 85% for the AMC [69,70]. These results contradictory to ours could be explained by the rational use of the ATB, the respect of the rules of breeding allowing to minimize the diseases and, at the same time, the use of ATB and an awareness of all the actors of the poultry sector (breeder, veterinarians, and state institutions) as to the need to preserve the existing ATB capital.

The results of antimicrobial resistance we got and which are quite disturbing can be explained by several factors; the increased and uncontrolled use of ATB, sometimes by the breeder himself, who is not, under any circumstances, entitled to perform correct antibiotic therapy. Indeed, nowadays, the large availability of avian ATB on the Algerian market with affordable prices (generic drugs) facilitates the access of these molecules to breeders [70]. The use of ATB as a preventive measure as growth promoters can also be incriminating, the non-respect of the rules of breeding such as increased density, bad hygiene, bad aeration, and the non-installation of footbath leads the appearance of pathologies involving repeated ATB therapies, in which antibiotherapy is carried out without using antibiograms in most of the time [75]. Other factors, mentioned by Mateo [76], may also influence the occurrence of resistance in poultry farms such as the decrease in the availability of ATB (poor dilution, degradation by biocides, and plugging pipettes), the decrease in consumption ATB (poor ATB taste, low number of water points, and lameness), and decreased absorption of ATB (enteritis). This phenomenon of antibioresistance can, also, be explained by the diversity of the mechanisms of resistance of bacteria [64].

### Antimicrobial activity of the EOs

As can be seen in Tables-6 and 7, the two EOs tested showed antibacterial activity against the strains tested; however, it was found that the essence of Thyme (*T. vulgaris* L.) had a much greater antibacterial activity than EO of Coriander (*C. sativum* L.). The average of the IZ recorded for the pure EO of Thyme on all the avian strains studied was estimated at 26.75±0.426 mm (ranging from 18.66±0.152 mm for the smallest IZ to 39.33±0.585 mm for the largest one) with CMIs ranging from 0.07 to 0.93 mg/ml; for this EO, it is noted that there is no variation between the average of the IZ of pure and diluted EO, and the same thing was observed by comparing the average of the IZ of broiler strains and those of turkeys.

**Table-6:** Antibacterial activity of the essential oils studied.

*Escherichia coli* strains	Broiler chicken strains (average of disc diameter of inhibition (mm)±standard deviation)			

	*Thymus vulgaris* L.		*Coriandrum sativum* L.	
	
	PEO	DEO	PEO	DEO
1	35.00±1.053	29.43±0.461	19.50±0.500	15.73±0.288
2	23.50±0.556	22.70±0.346	21.00±0.200	14.56±0.585
3	28.00±0.866	29.40±0.435	19.10±0.953	15.33±1.154
4	22.66±1.154	22.56±0.750	14.83±0.115	14.33±0.585
5	23.46±0.057	25.33±0.750	18.56±0.513	16.00±0.300
6	31.33±0.577	37.26±0.404	19.00±1.000	15.53±0.635
7	29.50±0.916	32.00±0.173	22.70±0.264	19.00±0.200
8	35.53±0.808	34.66±0.665	15.80±0.346	14.43±0.378
9	28.36±0.550	30.00±0.100	16.93±0.057	15.10±0.264
10	27.23±0.351	25.66±1.527	20.00±0.173	15.70±0.519
11	30.00±0.173	25.56±0.288	12.53±0.404	15.80±0.173
12	18.66±0.152	20.53±0.923	13.80±0.346	11.76±0.404
13	34.83±0.115	34.90±1.000	12.80±0.200	10.73±0.152
14	22.66±0.115	22.00±0.100	14.50±0.556	13.36±0.550
15	28.00±0.100	27.56±0.577	15.40±0.519	14.80±0.173
16	21.33±0.981	24.00±0.500	21.46±0.503	14.00±1.000
17	22.26±0.057	24.86±0.251	16.00±1.000	13.40±0.529
18	25.63±0.981	24.60±0.435	14.00±0.200	12.70±0.100
19	24.00±0.200	21.80±0.100	16.00±0.800	15.83±0.288
20	29.06±0.115	28.03±0.896	12.60±0.608	10.96±1.001
21	26.00±0.173	22.26±1.137	14.00±0.200	12.63±0.378
22	31.53±0.550	32.00±0.100	16.00±0.264	16.00±0.100
23	22.00±0.100	20.00±1.000	09.83±0.288	09.63±0.550
24	20.76±0.611	19.13±1.021	12.73±0.461	12.00±0.100
O.A.DDI-BCS	26.72±0.471	26.51±0.589	16.21±0.438	14.71±0.444

***Escherichia coli* strains**	**Turkey strains (average of disc diameter of inhibition (mm)±standard deviation)**			

	***Thymus vulgaris* L.**		***Coriandrum sativum* L.**	
	
	**PEO**	**DEO**	**PEO**	**DEO**

1	26.00±0.888	27.33±0.665	14.00±0.173	15.00±0.458
2	22.63±0.378	22.50±0.896	14.66±0.550	13.60±0.608
3	30.40±0.360	30.66±0.896	15.63±0.873	17.00±0.100
4	36.00±0.264	32.73±0.115	17.36±0.550	16.70±0.264
5	25.36±1.184	26.70±1.212	24.00±0.781	19.76±0.404
6	28.00±0.173	27.60±0.360	13.73±0.251	15.00±0.500
7	29.16±0.152	33.23±0.709	17.50±0.556	16.00±0.866
8	39.33±0.585	34.00±0.173	36.00±0.458	23.30±0.458
9	34.46±0.416	36.53±0.461	22.63±0.321	21.23±0.305
10	20.86±0.230	21.00±0.400	12.73±0.251	11.70±0.624
11	22.00±0.100	21.33±0.493	11.43±0.152	10.40±0.519
12	21.63±0.321	20.76±0.723	11.56±0.378	10.96±1.001
13	22.00±0.100	22.00±0.100	15.03±0.152	12.00±0.100
14	21.36±0.550	21.83±0.288	17.00±0.173	15.00±0.300
15	24.00±0.100	26.00±0.754	14.63±0.404	13.56±0.585
16	25.66±0.305	24.33±0.152	15.00±0.100	18.00±0.264
O.A.DDI-TS	26.80±0.382	26.78±0.496	17.05±0.383	15.57±0.461
O.A.DDI-AS	26.75±0.426	26.65±0.542	16.55±0.416	14.14±0.434
ATCC 25922	30.60±0.200	21.36±0.208	17.33±0.513	16.10±0.173

PEO=Pure essential oil, DEO=Dilute essential oil in 15% of DMSO, O.A.DDI-BCS=Overall average disc diameter of inhibition for broiler chicken strains, O.A.DDI-TS=Overall average disc diameter of inhibition for turkey strains, O.A.DDI-AS=Overall average disc diameter of inhibition for all strains, DMSO=Dimethyl sulfoxide

**Table-7:** MICs of the essential oils studied.

MIC of broiler chicken strains (mg/ml)		

*E. coli* strains	MIC TνEO	MIC C*s*EO
1	0.07	2.50
2	0.31	1.25
3	0.31	2.50
4	0.31	2.50
5	0.31	2.50
6	0.16	2.50
7	0.31	1.25
8	0.07	2.50
9	0.31	2.50
10	0.31	1.25
11	0.16	2.50
12	0.93	2.50
13	0.07	2.50
14	0.31	2.50
15	0.31	2.50
16	0.63	1.25
17	0.31	2.50
18	0.31	2.50
19	0.31	2.50
20	0.31	2.50
21	0.31	2.50
22	0.16	2.50
23	0.63	10
24	0.63	2.50

**MIC of turkey strains (mg/ml)**		

***E. coli* strains**	**MIC TνEO**	**MIC C*s*EO**

1	0.31	2.50
2	0.31	2.50
3	0.16	2.50
4	0.16	2.50
5	0.31	1.25
6	0.31	2.50
7	0.31	2.50
8	0.07	0.60
9	0.16	1.25
10	0.31	2.50
11	0.63	2.50
12	0.63	2.50
13	0.31	2.50
14	0.31	2.50
15	0.31	2.50
16	0.31	2.50
ATCC 25922	0.16	2.50

MIC=Minimum inhibition concentration, TνEO=*Thymus vulgaris* L. essential oil, C*s*EO=*Coriandrum sativum* L. essential oil, *E. coli=Escherichia coli*

After the Thyme EO, we found, with an average of IZ estimated at 17.05±0.383 mm (ranging from 9.63±0.550 mm for the smallest zone of inhibition to 36.00±0.458 mm for the largest one) and MICs evaluated between 0.6 and 10 mg/ml, the EO of Coriander. The aromatograms of this EO, on all avian strains studied, expressed different results between pure and diluted EO, with an estimated difference of 2.4 mm in favor of pure EO, a difference was also noticed between the broiler and turkey strains; in fact, turkey strains displayed, for both pure and diluted EO, an average of IZ larger, with a difference of almost 1mm, compared to the broiler chicken strains. Regarding the ATCC 25922, it expressed the same result regarding the sensitivity to the EOs used; Thyme EO in the first position with a MIC estimate at 0.16 mg/ml, followed by Coriander EO which the MIC had posted at 2.5 mg/ml. The IZ was greater in pure EO for the two EOs studied; in fact, for Thyme EO, a significant difference was highlighted between an IZ of 30.60±0.200 mm for pure EO versus 21.36±0.208 mm for diluted EO, for Coriander EO, a smaller gap has been registered between an IZ of 17.33±0.513 mm for pure EO against 16.10±0.173 mm for diluted EO, this result could be explained by an antagonism between the dispersant (DMSO) and these EO [3].

In the literature, several other studies have expressed results comparable to those we obtained regarding the antibacterial activity of Thyme EO [77-79] and Coriander EO [26,80] to *E. coli* ATCC 25922 and or multidrug-resistant *E. coli* strains. That said, in Egypt, a study by El-Shenawy *et al*. [81], rather revealed an antibacterial activity of Coriander EO (IZ=20 mm) greater than that we have found, and this contradictory result can be explained by the use of different bacterial strains of *E. coli*, different methods for MIC determination [82], the composition of EO, functional groups present in active component and their synergistic interactions, varietal differences, the test method used as well as culture conditions (type and volume of broth, temperature, time of incubation, concentration, and age of inoculums) [83-85].

In sum, it can be concluded that the antimicrobial properties of EO are essentially connected to their chemical composition; the highest antibacterial activity is demonstrated by phenolic compounds such as Carvacrol, Thymol, and Eugenol [86,87]. This would strongly explain the results we obtained; indeed, the most significant antibacterial activity reported was the one with the highest phenol levels, namely Thyme EO and its 74.40% phenolic compounds mainly represented by Carvacrol (73.03%). The mechanism of action of Carvacrol and Thymol, each on their side or in synergy, involves the disruption of the cell membrane and escape of cytoplasmic contents [88]. In *E. coli*, Carvacrol and Thymol provoke the depolarization and disintegration of the external membrane, liberating lipopolysaccharides and increasing the permeability of the cytoplasmic membrane [89,90]. Other studies, explaining even more the results we have obtained, rapported that, in addition to the ratio in which the main active constituents are present, the interactions between these and the minor constituents can also affect the antibacterial activity of EO; this is the case of *p*-cymen (whose level in Thyme were 9.99 %) which is not an efficient antimicrobial agent when used alone, but with Carvacrol and Thymol, it can potentiate the action of the EO to promoting the cytoplasmic membrane expansion and facilitating the antimicrobial action of these monoterpene phenols [90,91].

As for Coriander EO, its antibacterial activity was reported by several authors [92,93], this antibacterial activity can be explained by its chemical composition whose major component proved to be Linalool (60.91%) and its complex interactions with different individual components [94-96], the antibacterial activity in question would be due to disrupting bacterial cell walls, inhibiting bacterial enzyme activity and suppressing translation of certain regulatory gene products [92]. More recently, a new antimicrobial peptide, namely “plantaricin CS” with broad antibacterial activity was isolated from the coriander leaf extract [97].

## Conclusion

The present study has unfortunately confirmed the presence of an alarming ATB resistance, in Algeria, against *E*. *coli* in poultry farming, it has also demonstrated, *in vitro*, the antimicrobial action of two EOs, the EO of Thyme and that of Coriander, with a more pronounced antibacterial activity for Thyme EO, the results obtained are promising, *in vivo* study is necessary to validate the possibility of the use of EOs instead of ATBs.

## Authors’ Contributions

NM performed the fieldwork, collected the samples, isolated avian *E. coli* strains and determined their antimicrobial resistance profile, DH performed the aromatograms and highlighted the MICs, ND supervised the laboratory work, and LA read and approved the final manuscript. All authors have read and approved the final manuscript version.
